# Emancipation from transcriptional latency: unphosphorylated STAT5 as guardian of hematopoietic differentiation

**DOI:** 10.15252/embj.201693974

**Published:** 2016-02-18

**Authors:** Thomas Decker

**Affiliations:** ^1^ Max F. Perutz LaboratoriesUniversity of ViennaViennaAustria

**Keywords:** Immunology, Signal Transduction, Transcription

## Abstract

The canonical paradigm of Jak‐STAT signaling is that members of the signal transducers and activators of transcription (STATs) family of transcription factors are activated by Janus kinase (Jak)‐mediated tyrosine phosphorylation. While the relationship between activation and tyrosine phosphorylation still appears axiomatic, several lines of evidence suggest that unactivated, unphosphorylated isoforms, uSTATs, are nonetheless also engaged in transcriptional regulation. In this issue of *The EMBO Journal*, Park *et al* (2015) make a convincing case that nuclear uSTAT5 controls hematopoietic differentiation.

Jak‐STAT signaling was discovered in the 1990s as rapid two‐component signaling between cytokine receptors and cytokine‐regulated genes. Ligand‐activated receptor complexes contain active Jaks that phosphorylate one or more of the seven members of the STAT family on a single tyrosine residue. SH2 domain‐mediated dimerization then exposes nuclear import signals that relocate STATs to the nucleus. Target promoters for STAT dimers contain palindromic binding sites, designated gamma interferon‐activated site (GAS) after the prototypic element (Levy & Darnell, 2002). It has long been debated whether uSTATs exist in the nucleus with different results being obtained depending on the particular STAT and cell type (Reich & Liu, 2006). Initially, uSTATs were considered to be inactive precursors of the tyrosine‐phosphorylated pSTATs associated with “transcriptional latency”. This paradigm has been challenged by an increasing number of reports showing that cells employ uSTATs for diverse functions in the cytoplasm, mitochondria, and nucleus (Cheon *et al*, 2011; Gough *et al*, 2012; Sehgal, 2013). Green, Göttgens, and colleagues (Park *et al*, 2016) now report data underlining the importance of nuclear uSTATs. Importantly, the paper pinpoints exciting new prospects of their mechanism of action.

The STAT5 isoforms, STAT5a and STAT5b (collectively referred to as STAT5), are activated by multiple cytokine receptors, contributing to hematopoietic development. Gene‐targeted mice reveal important contributions of STAT5 to the generation of hematopoietic stem cells (HSC), committed progenitors, and their mature progeny (Wang & Bunting, 2013). Signaling by the thrombopoietin (TPO) receptor regulates HSC maintenance as well as megakaryocytic differentiation. TPO binding to its receptor activates STAT5. Park *et al* (2016) study STAT5 in a stem cell line that undergoes megakaryocytic differentiation in the presence of TPO. The cells are shown to contain nuclear uStat5, accumulating the tyrosine‐phosphorylated form (pSTAT5) in the nucleus only after TPO treatment. ChIP‐Seq data reveal that TPO causes a striking Stat5 redistribution within the cell genome. The authors identified three binding site clusters representing exclusive uSTAT5 binding, exclusive pSTAT5 binding, or sites for STAT5 association in both unstimulated and TPO‐treated cells. GAS sequences are highly represented in pSTAT5 binding regions, as expected. A novel and exciting finding is the occurrence of binding sites for CTCF in close proximity of roughly two‐thirds of the sites bound by uStat5 (Fig 1). CTCF is a DNA binding protein associated with both gene activation and gene repression, and has been reported to mediate chromatin interactions across distances. Importantly, the vast majority of uSTAT5 binding in the vicinity of CTCF (97%) is lost upon TPO treatment.

**Figure 1:**
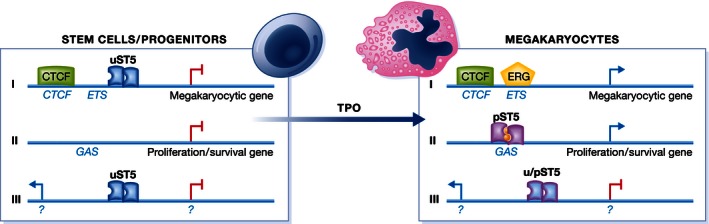
Unphosphorylated uSTAT5 and tyrosine‐phosphorylated pSTAT5 regulate different transcriptional programs in megakaryocytes and their parental stem cells This becomes manifest in three different STAT5‐regulated gene clusters. Cluster I is repressed in stem cells and characterized by CTCF and adjacent uSTAT5 binding sites. Cluster I genes are active in megakaryocytes when uSTAT5 disappears, giving way to ERG binding to ETS sequences. Cluster II genes contain pSTAT5‐specific binding sites (GAS). They are activated when TPO generates pSTAT5 dimers during megakaryocytic differentiation and include proliferation and survival genes. Cluster III contains genes associated with unchanged STAT5 binding before and after TPO treatment. These genes may be both repressed and active. It is unclear whether TPO treatment causes a uSTAT5‐pSTAT5 switch at cluster III gene promoters.

Genome regions with uSTAT5 and CTCF binding were highly enriched for genes involved in megakaryocyte and platelet development. In fact, the authors demonstrate that about 1,000 genes are affected by gene knockdown in the untreated stem cell line lacking detectable nuclear pSTAT5. About one‐third of these correspond to regions with uSTAT5 binding sites. STAT5 depletion provoked megakaryocytic differentiation. This is consistent with the observation that the same shRNA also causes increased expression of genes defining the megakaryocyte‐specific transcriptome. In summary, the data support the concept that uSTAT5 suppresses a transcriptional program required for megakaryocytic differentiation. The study further suggests that TPO/pSTAT5‐mediated redistribution to promoters with GAS sequences favors transcription of genes sustaining the survival of differentiating cells.

What is the mechanism behind gene repression by uSTAT5 and how is specificity achieved? Regarding the latter, the authors' unpublished evidence does not favor a direct CTCF–STAT5 interaction as a way of tethering uSTAT5 to chromatin. How binding occurs and to what sites remains open. The mechanism of repression is addressed with an additional data set including a comparison of global uSTAT5 binding with that of regulators of hematopoietic differentiation. It supports the view that uSTAT5 prevents binding of EGR, an activator promoting megakaryocytic differentiation. Similar to CTCF binding sites, EGR binding sites (ETS sequences) are highly represented in uSTAT5 binding regions. However, details of the mechanism behind the uSTAT5‐ERG antagonism remain to be clarified.

The strength of this paper is its convincing demonstration that a mammalian uSTAT regulates its own set of genes. Unlike other uSTAT activities (Cheon *et al*, 2011), uSTAT5 in fact behaves as a partial antagonist of biological pSTAT5 activity. The original demonstration that a uSTAT has widespread implications for a gene expression program and that tyrosine phosphorylation changes its impact derives from a combination of genetic and biochemical studies in *Drosophila* (Li, 2008). There, tumorigenesis is caused by constitutive activity of Hopscotch, the *Drosophila* Jak. The Hopscotch mutant disrupts heterochromatin. Consistently, unphosphorylated *Drosophila* STAT (STAT92E) contributes to heterochromatin formation and maintenance, and its absence from heterochromatin is associated with tumorigenesis and position effect variegation. The studies culminated in a model according to which a direct interaction between unphosphorylated uSTAT92E and heterochromatin protein 1 (HP1) stabilizes heterochromatin and gene silencing. Jak signaling thus affects the establishment and maintenance of heterochromatin by redirecting STAT92E to euchromatic binding sites. In favor of their model, the authors demonstrated a HP1 binding motif in STAT92E and, consistently, direct interaction between the proteins. As in the case of Park *et al* (2016), the molecular factors governing the association of uSTAT92E with chromatin remain to be determined. Whether a HP1‐dependent mode of action applies to STAT5, the vertebrate STAT most homologous to STAT92E was tested in mammalian cells. Indeed, interaction with HP1 was observed for overexpressed proteins and effects on heterochromatin and tumor formation reported (Hu *et al*, 2013). It should be noted, however, that the data in this study lack the elegance of the genome‐wide approaches applied by Park *et al* (2016). Moreover, an exclusive role of uSTAT5 in heterochromatin formation appears in disagreement with the fact that a large number of genes are positively controlled by the protein in hematopoietic stem cells. It remains an open question whether HP1 association, assuming it contributes to uSTAT5 action, is an alternative mode of action or related to the biological activity of uSTAT5 in hematopoietic stem cells.

An urgent question posed by the study in hematopoietic stem cells concerns the implications of uSTAT5 binding in the vicinity of the transcription factor CTCF. Conceivably, CTCF might be involved in uSTAT5 association with DNA or, vice versa, uSTAT5 might impact on the transcriptional activity of CTCF. CTCF has diverse impact on gene transcription, much of which is through the establishment of long‐range chromatin interactions (Merkenschlager & Odom, 2013). Many of these require interaction with cohesins. However, CTCF also mediates promoter–enhancer interaction independently of cohesins (Ball *et al*, 2014). Of importance in light of uSTAT5‐mediated suppression of megakaryocyte genes, CTCF is able to block the interaction between enhancers and promoters. It remains to be determined whether uSTAT5 colocalizes with cohesins and how it affects the surrounding chromatin landscape. Genome‐wide chromatin conformation capture technology will allow to study the impact of uSTAT5, and the changes set off by TPO‐activated pSTAT5, on 3D interactions of the stem cell genome.

Deletion of STAT5 in mouse hematopoietic stem cells (HSC) strongly impairs their fitness and ability for competitive repopulation of hematopoietic cells (Wang & Bunting, 2013). Whether and how expressing a tyrosine‐phenylalanine mutant, that is, constitutive uSTAT5, would alter the long‐term survival of HSC, and to which extent these cells could give rise to hematopoietic progeny is an open question. Experimentally, it may be challenging however to derive HSC expressing phenylalanine substitutions for both STAT5 genes and to interpret their behavior in light of the multitude of hematopoietic functions of STAT5.

Finally, it is tempting to reflect on the data presented by Park *et al* (2016) from an evolutionary perspective. Did uSTAT activity coevolve with, or even precede, the transcriptional activity of pSTATs? *Dictyostelium* is a well‐studied simple organism containing STATs (Kawata, 2011). While *Dictyostelium* STATs evolved without a *bona fide* transactivating domain they all contain a tyrosine subject to phosphorylation. In fact, all STAT‐containing organisms preceding the establishment of a Jak–STAT pathway in most of bilaterian animals use different tyrosine kinases to activate STATs. From this perspective, it appears unlikely that uSTATs preceded pSTATs. This does not exclude, however, that STATs evolved the ability to function in absence of tyrosine phosphorylation in parallel to their pSTAT activities. Studies such as the one now presented by Park *et al* (2016) may spark efforts to place this hypothesis under scientific scrutiny. Studies in organisms representing different branches of the evolutionary tree may help to clarify whether a key finding of this paper, the existence of different target genes for uSTAT and pSTAT, is paradigmatic. Likewise, such studies will reveal whether controlling distinct sets of genes is a common denominator of all members of the vertebrate STAT family, or reserved to a subset. In all likelihood, future research will produce more and exciting new insight into the uSTAT–pSTAT relationship.
